# British Ornithology

**Published:** 1811-04

**Authors:** 


### BRITISH ORNITHOLOGY.

The third number of this interesting publication contains Larus Carus, the common Gull, a spirited drawing. Mr. Graves has abstained from giving any other specific characters than the length, breadth, and weight, as these birds vary so much in plumage; there wants a series of correct figures, such as this work seems likely to produce, to illustrate the subject; we suggest the propriety of giving figures of each species at the different periods of their age when likely to mislead the unscientific enquirer. Anas Nigra, the Scoter; of this species we do not recollect a good figure in any preceding author, the principal distinguishing character is the protuberance at the base of the bill, which is here very accurately defined; we learn that they are supposed to breed in the Calf of Man, and that further information on this head will be given at a future period. Motacilla Troglodytes, common Wren; this lively little aminal is admirably depicted; we regret the figure is so small, and would recommend the author in future to give the figures on a larger scale, more particularly as he constantly gives the length, breadth, and weight, which will do away any false idea of size. Fringilla Carduelis, the goldfinch, a pretty good figure, we think on the whole, the bird is rather clumsy; the description is interesting. Picus viridis, the green Woodpecker; the draftsman has been very happy in delineating this picturesque bird, as its modes of support and manner of feeding are well expressed, the history is particularly interesting. Anas Boschas, the wild Duck; this figure is equal to any given, and forms a more correct portrait of this most elegant species than any former work contains, the descriptive part is good and the history entertaining; upon the whole we consider this superior to the former numbers.

